# Concise N-doped Carbon Nanosheets/Vanadium Nitride Nanoparticles Materials via Intercalative Polymerization for Supercapacitors

**DOI:** 10.1038/s41598-018-21082-w

**Published:** 2018-02-13

**Authors:** Yongtao Tan, Ying Liu, Zhenghua Tang, Zhe Wang, Lingbin Kong, Long Kang, Zhen Liu, Fen Ran

**Affiliations:** 1 State Key Laboratory of Advanced Processing and Recycling of Non-ferrous Metals, Lanzhou University of Technology, Lanzhou, 730050 P. R. China; 2 School of Material Science and Engineering, Lanzhou University of Technology, Lanzhou, 730050 Gansu P. R. China; 3 Department of Physics & Engineering, Frostburg State University, Frostburg, MD 21532-2303 USA; 4 Guangzhou Key Laboratory for Surface Chemistry of Energy Materials, New Energy Research Institute, School of Environment and Energy, South China University of Technology, Guangzhou Higher Education Mega Centre, Guangzhou, 510006 China; 5 Guangdong Provincial Key Laboratory of Atmospheric Environment and Pollution Control, Guangdong Provincial Engineering and Technology Research Center for Environmental Risk Prevention and Emergency Disposal, South China University of Technology, Guangzhou Higher Education Mega Centre, Guangzhou, 510006 China; 6 Department of Chemistry, Xavier University of Louisiana, New Orleans, LA 70125 USA

## Abstract

N-doped carbon nanosheets/vanadium nitride nanoparticles (N-CNS/VNNPs) are synthesized via a novel method combining surface-initiated *in-situ* intercalative polymerization and thermal-treatment process in NH_3_/N_2_ atmosphere. The pH value of the synthesis system plays a critical role in constructing the structure and enhancing electrochemical performance for N-CNS/VNNPs, which are characterized by SEM, TEM, XRD, and XPS, and measured by electrochemical station, respectively. The results show that N-CNS/VNNPs materials consist of 2D N-doped carbon nanosheets and 0D VN nanoparticles. With the pH value decreasing from 2 to 0, the sizes of both carbon nanosheets and VN nanoparticles decreased to smaller in nanoscale. The maximum specific capacitance of 280 F g^−1^ at the current density of 1 A g^−1^ for N-CNS/VNNPs is achieved in three-electrode configuration. The asymmetric energy device of Ni(OH)_2_||N-CNS/VNNPs offers a specific capacitance of 89.6 F g^−1^ and retention of 60% at 2.7 A g^−1^ after 5000 cycles. The maximum energy density of Ni(OH)_2_ ||N-CNS/VNNPs asymmetric energy device is as high as 29.5 Wh kg^−1^.

## Introduction

Electrochemical capacitors (ECs), also called supercapacitors or ultra-capacitors, has caught the eye of many and is considered to be a promising energy storage candidate due to its fast charging and discharging rates in seconds, high power density, long cycle life, etc. Furthermore, ECs bridges the gap between battery and traditional electric capacitors, which is widely applied in hybrid electrical vehicles, energy-storage systems, and portable micro-electronic devices^1–4^. In general, the typical ECs are divided into two categories: electric double layer capacitors (EDLCs), which store energy on the basis of charges absorbed at the surface or interface of electrode/electrolyte; and pseudocapacitors which store energy based on redox-reaction (faradic reaction) occurring at the surface of electrode. In this case, electrode materials played a key role to determine the electrochemical performance of supercapacitors^5^.

Many kinds of electrode materials have been developed for supercapacitors. Carbon-based materials, such as carbon nanotubes^6^, ordered mesoporous carbon^7^, hierarchical porous carbon^8^, biomass carbon^9–11^, N-riched carbon^12,13^, and graphene^14^ *et al*., show standard characteristics of EDLCs, however, the relatively low specific capacitance and energy density (<10 Wh kg^−1^) hinder the practical application. Meanwhile, various kinds of metal oxides or hydroxides, such as VO_x_^15,16^, MnO_2_^17–19^, Co_3_O_4_^20–23^, NiO^24–27^, Ni(OH)_2_^28–30^, and Co(OH)_2_^31,32^, *et al*., and conducting polymers, such as polyaniline^33–36^ and polypyrrole^37^, *et al*., have been developed as pseudocapacitive materials. Although these pesudocapacitive materials exhibit high specific capacitance, they all suffer from the same problems which include poor electrical conductivity and a relatively poor cycle life. In addition, most metal oxides are cathode materials or positive materials.

Compared to metal oxides/hydroxides and conducting polymers, transition metal nitrides like Molybdenum Nitride (MoN), Titanium Nitride (TiN), Vanadium Nitride (VN), and Tungsten Nitride (WN)^38–40^ etc. have numerous advantages. For example, transition metal nitrides exhibit considerable voltage range, good sustainability, and high electrical conductivity. Among them, VN, has and continues to be widely investigated by Choi *et al*. for supercapacitors as anode materials. The highest specific capacitance value of 1340 F g^−1^ at 2 mV s^−1^ was achieved and 554 F g^−1^ was attained at a higher scan rate of 100 mV s^−1^ between −1.2 and 0 V^41^. The super high specific capacitance was caused by the high conductivity and nanoscale size of the thin oxide that existed on the surface of VN nanocrystal. Zhou *et al*. reported VN synthesized by calcining V_2_O_5_ xerogel, delivering a specific capacitance of 161 F g^−1^, in 1 M KOH^42^. The VN nanoparticles ranging from 15 nm to 110 nm achieved a specific capacitance of 186 F g^−1^ in 1 M KOH at 1 A g^−1^ reported by Glushenkov *et al*.^43^. Lu *et al*. synthesized a porous VN nanowire on a carbon cloth, which showed a specific capacitance of 298.5 F g^−1^ in 5 M LiCl^44^. Xu *et al*. synthesized VN nanofibers via a combination of electrostatic spinning and high-temperature calcination in ammonia, which exhibited high specific capacitance of 291.5 F g^−1^^45^. Recently, VN quantum dot/nitrogen-doped microporous carbon nanofibers were further developed by the same method of combination between electrostatic spinning and high-temperature calcination under the mixed atmosphere in our previous work. The fabricated VN quantum dot/nitrogen-doped microporous carbon nanofibers showed a high specific capacitance of 406.5 F g^−1^^46^, which highlighted the merit of 1D continuous VN nanofibers. More recently, VN/nitrogen-doped graphene composites were fabricated, showing a high specific capacitance of 445 F g^−1^ proving to be a stable high performance anode material for supercapacitors^47^. Meanwhile, Wang *et al*. reported the hybrid 2D to 0D graphene–VN quantum dots for superior lithium and sodium storage with a good performance^48^. These two works highlighted the merits of a combination between 2D and 0D architecture as electrode materials for energy storage application. In this case, a simple method and strategy needs to develop to fabricate 2D vanadium-based materials as anode electrode materials for supercapacitors.

In this paper, a simple method combining surface-initiated *in-situ* intercalative polymerization of aniline and thermal-treatment process was used to fabricate supercapacitor anode material of N-doped carbon nanosheets/VN nanoparticles materials. The surface-initiated reaction was carried out on the surface of V_2_O_5_ dry xerogel, which used as both soft templates and oxidative agents, as well as 2D sheet-like morphology. The simple but novel method provided the combination of the 2D and 0D nanostructure. The N-doped carbon nanosheets and super small VN nanoparticles exhibited high specific capacitance of 424 F g^−1^, and nice energy density of 29.5 Wh kg^−1^, when assembled as an energy device of Ni(OH)_2_||N-CNS/VNNPs.

## Experimental Section

### Chemicals

Ammonium metavanadate (NH_4_VO_3_) and hydrogen peroxide (H_2_O_2_, 30%) were purchased from Tianjin Guangfu Technology Development *Co., Ltd*., and Tianjin HengXing Chemical Reagent *Co. Ltd*., which were used as received. Aniline was purchased from Sinopharm Chemical Reagent *Co. Ltd* and distilled prior to use.

### Preparation of V_2_O_5_ Dry Xerogel

i) NH_4_VO_3_ was annealed in an air atmosphere at 500 °C for 2 h; ii) 2 g of the obtained V_2_O_5_ powder was mixed with 10% of H_2_O_2_ (180 mL), which was stirred at ~0 °C for 6 h; iii) the mixture was kept standing at room temperature for 3 days; iv) V_2_O_5_ dry xerogel was obtained by drying at 100 °C in a drying oven for evaporation of solvent.

### Synthesis of V_2_O_5_ Dry Xerogel/Polyaniline Intercalative Hybrids

0.1 g of V_2_O_5_ dry xerogel was dispersed into 40 mL of HCl aqueous solution (pH = 0, 1, or 2) under ultrasonic treatment for 10 min and stirring 5 min. Meanwhile, 0.05 g of aniline monomer was dissolved into 20 mL of HCl aqueous solution. The two fresh prepared solutions were then rapidly mixed and vigorously shaken for 30 min, which was kept standing at room temperature for 24 h. After that, the dark-green precipitation was centrifuged at the speed of 8000 r min^−1^ and washed with deionized water and ethanol alternatively for three cycles. V_2_O_5_ dry xerogel/polyaniline intercalative hybrids were obtained by drying them at 60 °C for 24 h.

### Fabrication of N-doped Carbon Nanosheets/Vanadium Nitride Nanoparticles (N-CNS/VNNPs) Materials

N-doped carbon nanosheets/vanadium nitride nanoparticles hybrids were fabricated by annealing V_2_O_5_ dry xerogel/polyaniline intercalative hybrids at 700 °C for 2 h in a tube furnace with the mixing gas atmosphere of N_2_ and NH_3_ (N_2_:NH_3_ = 40:100) at a heating rate of 5 °C/min. For comparison, normal vanadium nitride was prepared by annealing V_2_O_5_ dry xerogel in the same condition.

### Materials Characterization

The morphologies were examined by scanning electron microscopy (SEM, JSM-6701) and transmission electron microscopy (TEM, TECNAI TF20). The crystal structures and compositions of the as-prepared samples were characterized by using X-ray diffraction (XRD, D/max-2400, Rigaku, Cu Kα, 0.154056 nm). X-ray photoelectron spectra (XPS) were recorded on a Perkin-Elmer PHI ESCA system. Specific surface areas were measured by Brunauer-Emmett-Teller (BET) nitrogen adsorption–desorption (Micromeritics ASAP 2020 Instrument, USA) and pore-size distributions were calculated by the Barrett-Joyner-Halenda (BJH) method using the desorption branch of the isotherm.

### Electrochemical Performance

#### Electrode Preparation

N-doped carbon nanosheets/vanadium nitride nanoparticles hybrids, conducting graphite, acetylene black, and poly (tetrafluoroethylene) at the weight ratio of 80:7.5:7.5:5, were mixed in an agate mortar, which was coated on the Ni foam of geometric surface area of *ca*. 1 cm^2^ as current collector. After being pressed at 10 M Pa for *ca*. 15 s, finally, the electrode was obtained by being dried at 80 °C for 12 h. In all of the electrodes, the mass loading of active materials was of 2 mg cm^−2^

#### Electrochemical Performances

The electrochemical evaluation was measured by a three-electrode system including the N-doped carbon nanosheets/vanadium nitride nanoparticles hybrids as the working electrode, a platinum foil electrode as counter electrode, and a saturated calomel electrode (SCE) as reference electrode. Cyclic voltammetry (CV) and galvanostatic charge/discharge (GCD) measurements were tested in a 2 M KOH aqueous solution. Electrochemical impedance spectroscopy (EIS) measurements were performed over a frequency in a range of 0.01~10^5^ Hz at an amplitude of 5 mV. Cycle stability was tested by using CT2001A (Land, China). All the electrochemical experiments were carried out at 20 ± 1 °C and using the CHI660E workstation.

Specific capacitance (SC) was calculated from GCD curve according to equation:

1 $${C}_{s}=I\Delta t/m\Delta V$$

where *C*_*s*_ is specific capacitance (F g^−1^), *I* is discharging current (A), *Δt* is discharging time (s), *m* is active material mass (g), and *ΔV* is potential window (V).

### Preparation of Supercapacitor Device

The energy device was fabricated with Ni(OH)_2_ as the positive electrode, and obtained an active material as the negative electrode. For the energy device, the charge balance follows the relationship:

2 $${Q}_{+}={Q}_{-}$$

Where *Q* represents the charge stored, “+” and “−” represent positive electrode and negative electrode, respectively. Knowing that charge, Q, is equal to current, I, multiplied by time, t, we can rearrange equation 1 to be

3 $$Q={C}_{s}\times m\times \Delta V$$

According to above equations (2) and (3),

4 $${m}_{+}/{m}_{-}={C}_{s-}/{C}_{s+}\times \Delta {V}_{-}/\Delta {V}_{+}$$

The energy density (E) and power density (P) were calculated based on the following equations:

5 $$E=0.5{C}_{s}\Delta {V}^{2}$$

6 $$P=E/t$$

## Results and Discussion

**Figure 1:**
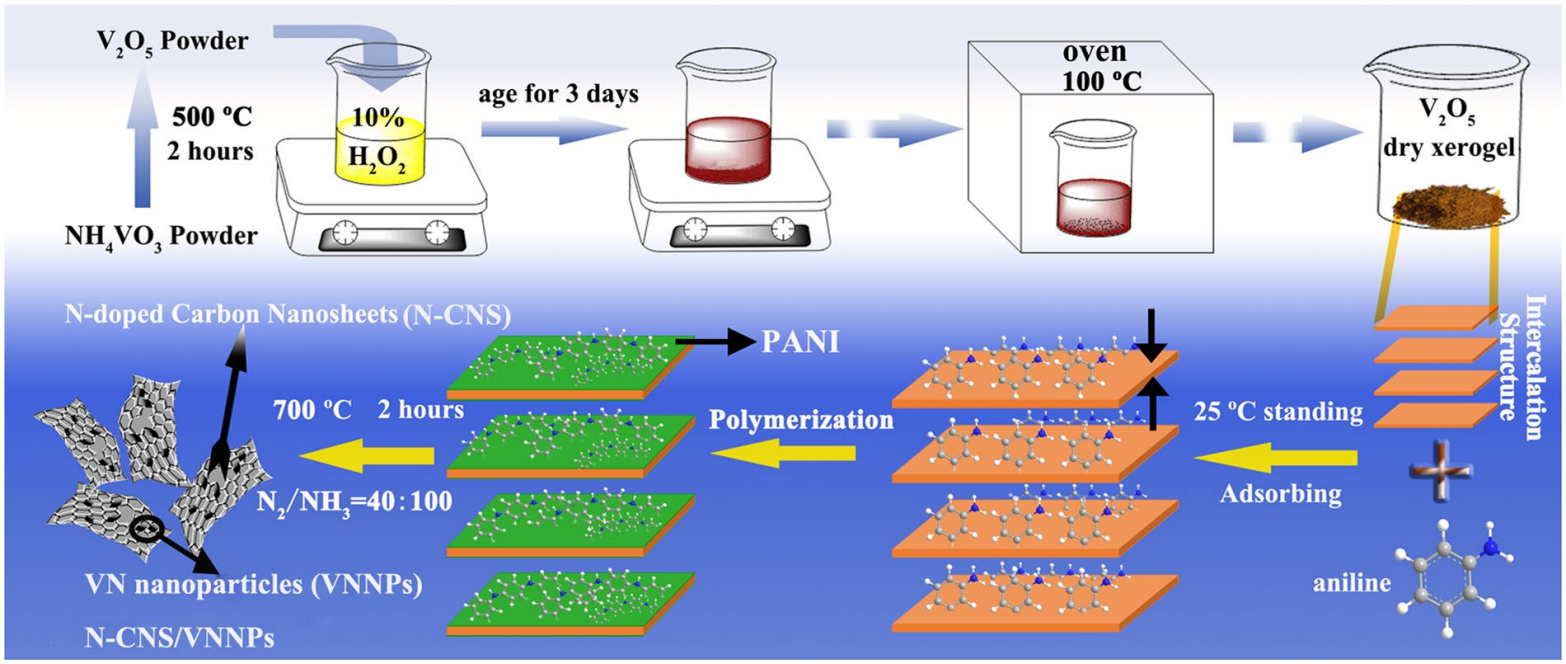
Schematic illustration of the synthesis process of N-CNS/VNNPs hybrids.

The fabrication process mainly included preparation of V_2_O_5_ dry xerogel with intercalation structure, synthesis of V_2_O_5_/polyaniline by surface-initiated *in-situ* intercalative polymerization, and subsequently thermal-treatment, which was depicted in detail in the experimental section and can be briefly illustrated in Fig. 1. Through several steps like annealing, reacting with H_2_O_2_, and evaporating solvent, commercial NH_4_VO_3_ powder was transformed to V_2_O_5_ dry xerogel. It should be pointed out that the step of aging for 3 days was necessary to ensure thorough reaction of V_2_O_5_ powder with H_2_O_2_, where the color of the mixture solution changed from light yellow to dark red. Take into account these technologies, V_2_O_5_ dry xerogel would show an intercalation structure, and thus aniline could easily be accessed and adsorbed to the surface of V_2_O_5_ dry xerogel by electrostatic force or Vander Waals’ force. After that, polymerization of aniline was launched on the layer of V_2_O_5_ dry xerogel. V_2_O_5_ acted as both the oxidative agent of aniline polymerization and the soft template of structural evolution. Note that V_2_O_5_ dry xerogel would also be etched during the surface-initiated *in-situ* intercalative polymerization. By using the intercalative V_2_O_5_ dry xerogel as a soft template, polyaniline (PANI) can be prepared into the structure of nanosheets. Meanwhile, the V_2_O_5_ layer would be largely consumed and small amounts of V_2_O_5_, in nanoscale, would be independently left on the surface of the PANI nanosheets. Finally, N-doped carbon nanosheets/vanadium nitrogen nanoparticles (N-CNS/VNNPs) would be obtained by the annealing process in the mixed gas atmosphere.

**Figure 2:**
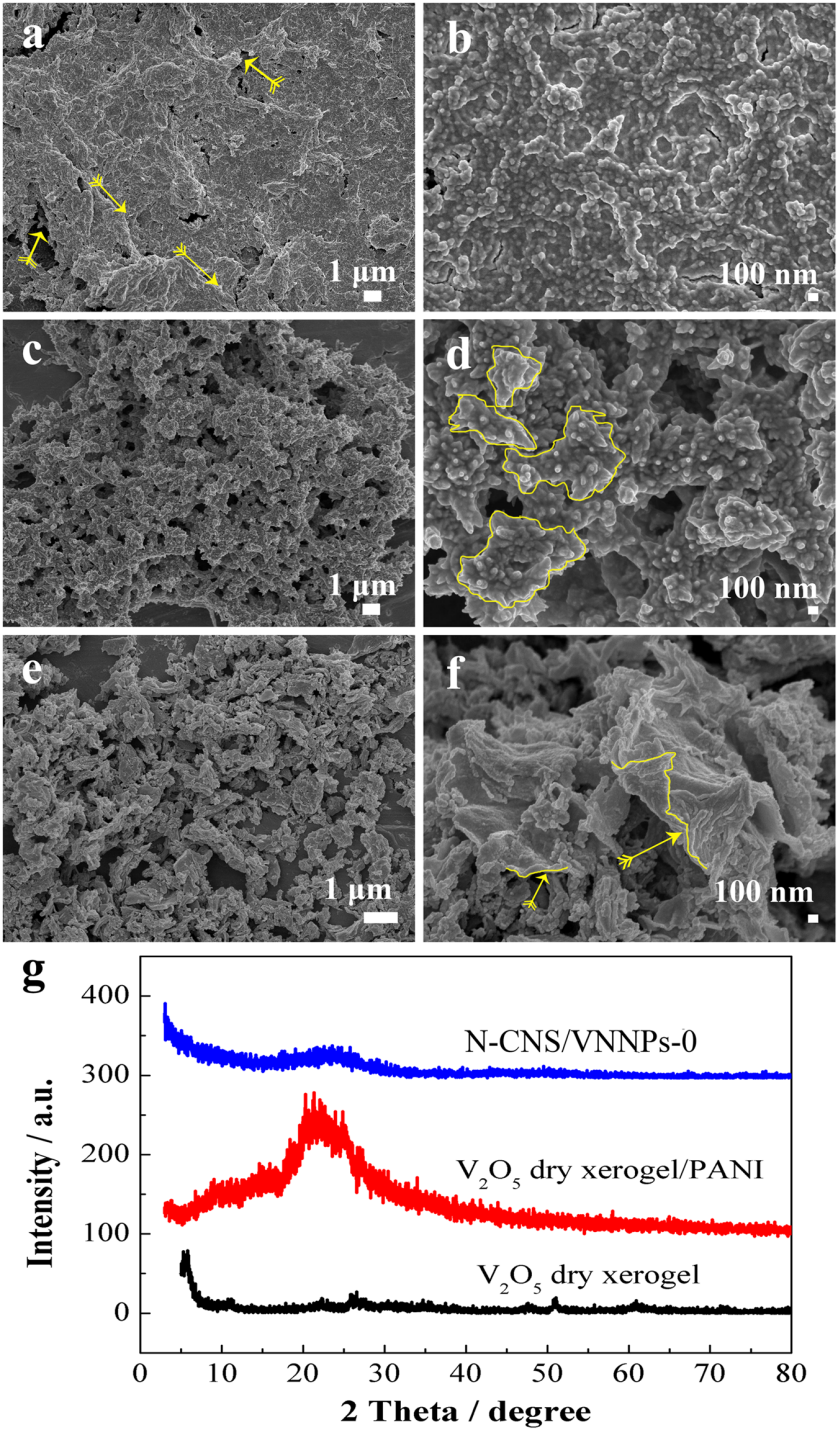
SEM images of (**a** and **b**) V_2_O_5_ dry xerogel, (**c** and **d**) V_2_O_5_ dry xerogel/polyaniline intercalative hybrids, (**e** and **f**) N-CNS/VNNPs, and (**g**) XRD patterns.

The corresponding SEM images and XRD patterns for V_2_O_5_ dry xerogel, V_2_O_5_ dry xerogel/polyaniline, and N-CNS/VNNPs are shown in Fig. 2. V_2_O_5_ dry xerogel exhibited wrinkled morphology consisting of nanoparticles, which were close-packed together. The V_2_O_5_ dry xerogel looked like stacking by a lot of sheets (Fig. 2a and b); however, V_2_O_5_ dry xerogel/polyaniline hybrid showed small, irregular and loose sheet-like shape consisting of nanoparticles or nano-short rods attached on the surface (Fig. 2c and d). This is because V_2_O_5_ xerogel were dissolved partly in the strong acid solution (pH = 0) so that a lot of small sheets formed for V_2_O_5_/PANI hybrids. PANI nanosheets formed due to the sacrifice of the template of V_2_O_5_ and the surface-initiated *in-situ* polymerization of aniline in the interval between layers. N-CNS/VNNPs hybrid as shown in Fig. 2e and f, exhibited wrinkled nanosheets with nanoparticles derived from nanosheets of V_2_O_5_ dry xerogel/PANI hybrids including nanoparticles on the surface. This is because in the heat-treatment process, PANI was carbonated, V_2_O_5_ phase was translated to VN phase, and meanwhile, the layers could be exfoliated to form the nanosheets with nanoparticles.

The XRD patterns are shown in Fig. 2g. There were weak diffraction peaks of V_2_O_5_ dry xerogel at 2θ values of ~12°, ~27°, ~51°, and a strong diffraction peak at ~5.6°, exhibiting the amorphous intercalated structure of V_2_O_5_^49^. After polymerization, the broad diffraction peak of V_2_O_5_ dry xerogel/PANI hybrids at ~22° was the characteristic sign of PANI intercalation^50^. After further annealing, a broad diffraction peak of N-CNS/VNNPs at ~23° was indexed as crystal planes of (002) of amorphous carbon^48^. Interestingly, there was no obvious VN crystal peak, indicating small amounts of VN materials left in the hybrid and relatively poor crystallinity, which would contribute much to improve the charging storage during charging and discharging process.

**Figure 3:**
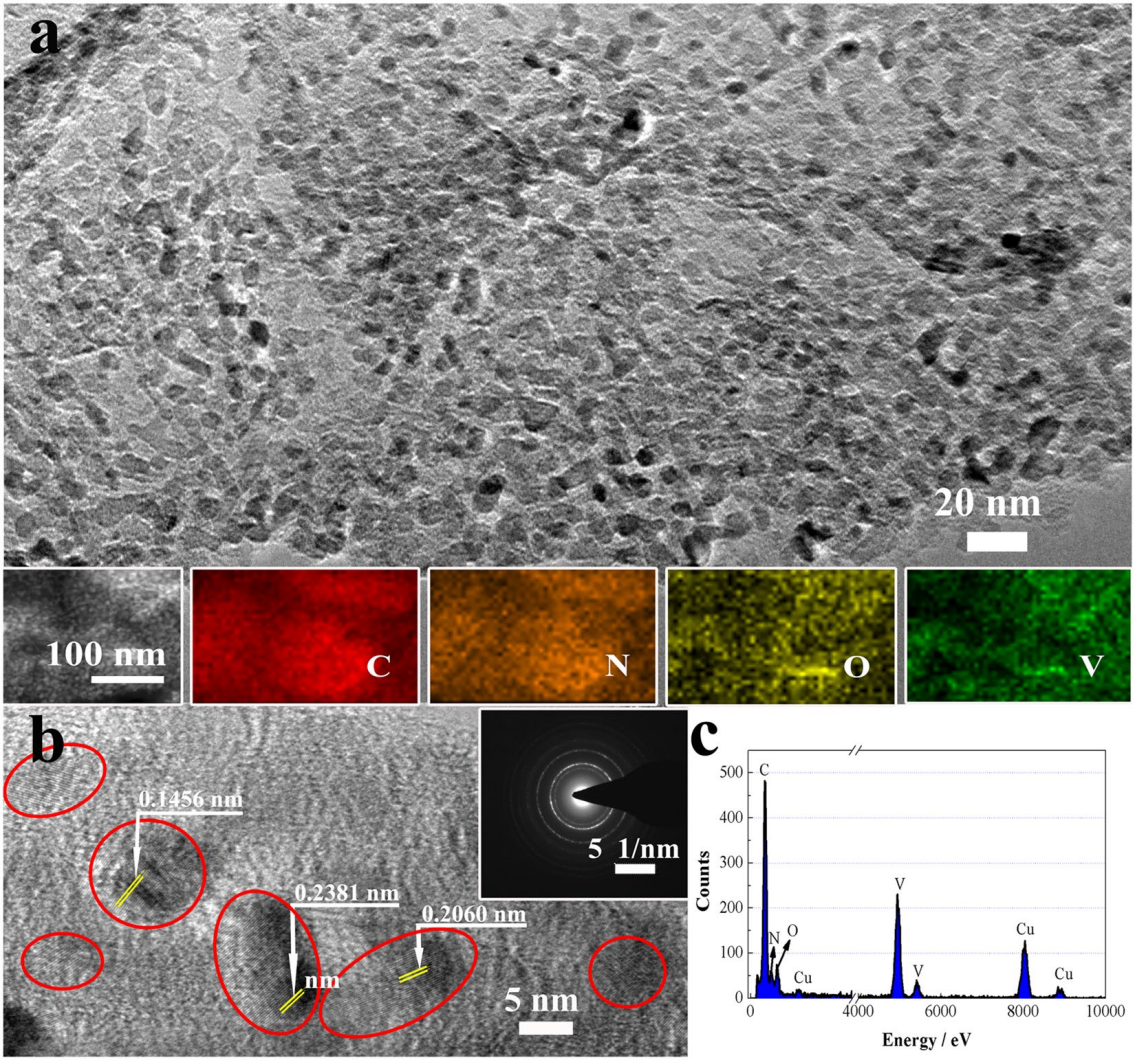
(**a**) TEM image (inset: EDS elemental mapping), (**b**) HRTEM image (inset: SAED), and (**c**) EDS data of N-CNS/VNNPs.

N-CNS/VNNPs exhibited sheet-like shape of carbon and nano size of VN, which was further confirmed by TEM, HRTEM, and EDS methods, as shown in Fig. 3 and Figures S1~S2 in the Supporting Information. From Fig. 3a and Figure S1 in the Supporting Information, one can see that the nanosheets were pretty thin and uniform, and contained nanoparticles on the surface. From HRTEM characterization in Fig. 3b, the crystal lattice was founded and marked. The lattice fringes with interplanar spacings of 0.2381, 0.2060 and 0.1456 nm corresponded to the (111), (200) and (220) planes of cubic VN (JCPDS NO. 73-0528), respectively. Inset photo in Fig. 3b showed the selected area electron diffraction (SAED), revealing that VN was polycrystalline. Figure 3c plotted EDS of N-CNS/VNNPs hybrids, representing the components of V, N, O, and C elements. This result agreed well with that of EDS elemental mapping (inset photos in Fig. 3a). In addition, uniform distribution of the elements of V, N, O, and C revealed the facts of N doping in carbon nanosheets and O existence in the surface of VNNPs.

**Figure 4:**
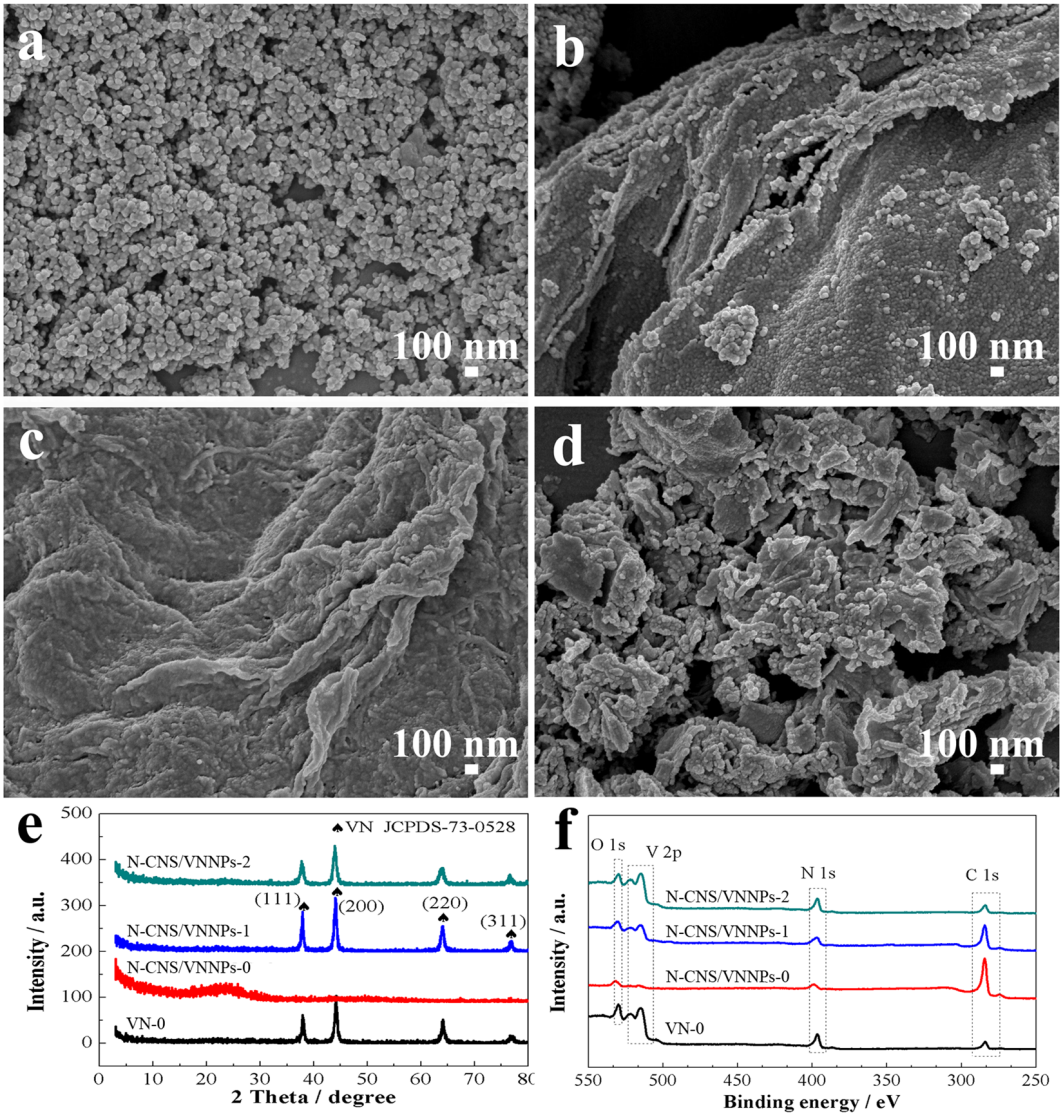
SEM images of (**a**) VN-0, (**b**) N-CNS/VNNPs-2, (**c**) N-CNS/VNNPs-1, (**d**) N-CNS/VNNPs-0; (**e**) XRD patterns, and (**f**) XPS spectra.

It is well known that pH value is very important for preparation of PANI^51^; more importantly, in this work, pH value also has a critical effect on solubility of V_2_O_5_ dry xerogel in the reaction system, which acted as a reactive template. Because of the different dissolution to varying extent, the photos of samples in different ultrasonic time are shown in Figure S3, it is noted that even if at the strong acid condition (pH = 0) with ultrasonic condition, the V_2_O_5_ xerogel could be dissolved, but need longer time than that in 20 minutes. However, at the conditions of pH = 1 or pH = 2, V_2_O_5_ xerogel could be only dissolved a little bit. In our experimental process, the time control appears very important. The morphologies of the obtained N-CNS/VNNPs hybrids should be completely different. In this case, N-CNS/VNNPs hybrids were synthesized at different pH values of 0, 1, and 2 during surface-initiated *in-situ* intercalative polymerization process, which were termed as N-CNS/VNNPs-0, N-CNS/VNNPs-1, and N-CNS/VNNPs-2, respectively. For comparison, normal VNs were prepared by annealing NH_4_VO_3_ and V_2_O_5_ dry xerogel in the same condition and termed as VN and VN-0, respectively. The morphologies and structure information of VN-0, N-CNS/VNNPs-0, N-CNS/VNNPs-1, and N-CNS/VNNPs-2 were identified by SEM, XRD, and XPS, as shown in Fig. 4. Both VN and VN-0 consisted of particles relatively on a larger scale (see Fig. 4a and Figure S4 in Supporting Information), while all of the N-CNS/VNNPs samples were 2D to 0D carbon nanosheets with embedded VN nanoparticles on its surface. Moreover, when pH value decreased from 2 to 0, carbon sheets became thinner and smaller in nanoscale, and VN nanoparticles became less and unexposed. This is because the lower pH value made V_2_O_5_ dry xerogel dissolve better to show the specific intercalation structure and surface-initiate *in-situ* intercalation polymerization^52,53^. Figure 4e showed XRD patterns. Four strong characteristic diffraction peaks of VN were observed at the 2θ value of 37.6°, 43.8°, 63.6°, and 76.4° in the spectrum of VN-0, which can be ascribed to the crystal planes of (111), (200), (220), and (311), respectively, which is consistent with cubic VN crystal (JCPDS NO. 73-0528). N-CNS/VNNPs-2 and N-CNS/VNNPs-1 exhibited the same cubic VN as VN-0 but no diffraction peak at 2θ value of ~23° which indicated a lower carbon content; however, N-CNS/VNNPs-0 showed the board diffraction peak at 2θ value of ~23° ascribed to the high amount of amorphous carbon. With the pH value increasing, the diffraction peaks of VN become obvious due to the increase of VN content and size in the samples, which were consistent with SEM results. XPS characterization for VN-0, N-CNS/VNNPs-0, N-CNS/VNNPs-1, and N-CNS/VNNPs-2 are shown in Fig. 4f and Figure S5–8in Supporting Information. V 2p^3^ line was fitted three peaks at 513.8, 515.5 and 517.1 eV which belongs to V^3+^ in VN, V^4+^, and V^5+^ in oxidized vanadium, respectively^43^. Oxidized vanadium was further demonstrated by the high-resolution O 1 s peaks at 530.1 eV for O–V bond, ascribed to the easy oxidation of the VN surfaces^48^. While the pH value increased, the contents of both V and N increased, while the content of carbon decreased. The detailed compositions of these samples are shown in Table 1. It could be seen that when pH values were 2, 1, and 0, the contents of vanadium were 26.91, 10.25, and 1.58 at.%, while carbon contents were to 27.72, 62.62, and 83.78 at.%, respectively. This phenomenon again confirmed the sacrifice of V_2_O_5_ and the formation of residual VN nanoparticles at a lower pH condition. In addition, the atom contents of V and N were 28.18 and 28.49 at.%, respectively, the ratio of which was near to 1:1. Interestingly, the atom ratios of N to V in N-CNS/VNNPs-2, N-CNS/VNNPs-1, and N-CNS/VNNPs-0 were 1.08, 1.55, and 5.55, respectively. This also confirmed that it was easier for surface-initiated *in-situ* intercalative polymerization when the pH value was 0. Besides, the N that came from VNNPs, an observable amount of N was doped in carbon bulk. The high resolution of N 1 s line was fitted into two peaks at 396.8, and 399 eV for VN-0 (Figure S5)^46^, however, the N 1 s line of N-CNS/VNNPs-0 was fitted into four peaks at 398.5, 400.1, 401.0, and 403.3 eV (weak peak), belonging to N-V in VN, pyrrolic (N-5), graphitic (N-Q) nitrogen, and N-O, respectively^54^ (Figure S6 in Supporting Information). The N 1 s lines of N-CNS/VNNPs-1 was fitted for four peaks at 396.5 and 398.5, belonging to N-V in VN, and 400.4 and 403.2 eV (weak peak and little content) credited to pyrrolic (N-5) and N-O, respectively (Figure S7 in Supporting Information). The N 1 s lines of N-CNS/VNNPs-2 was fitted for four peaks at 396.8 and 398.9, belonging to N-V in VN, 401.3 and 403.2 eV (weak peak and little content) ascribed to graphitic (N-Q) nitrogen and N-O, respectively (Figure S8 in Supporting Information). Obviously, the additional two peaks (N-5 and N-Q) of the N 1 s line of N-CNS/VNNPs were observed. The carbon nanosheets were doped with nitrogen, which can improve conductivity and penetrating quality in electrolyte benefiting to electrochemical reaction kinetics^12,13^ and N-doped fractions (% N-5 + N-Q) which were 37.1, 5.0, and 3.78% for N-CNS/VNNPs-0, N-CNS/VNNPs-1 and N-CNS/VNNPs-2, respectively. In summary, formation of the nanosheets structure, N doping of carbon bulk, and small amount of VN nanoparticles would contribute significantly to the enhancing of the conductivity of the electrode, easily moving and touch with electroactive material surface of ions, electrolyte-affinity of the electrode, and further improving the electrochemical performance.

**Table 1:** XPS results for the surface compositions of VN-0, N-CNS/VNNPs-2, N-CNS/VNNPs-1, and N-CNS/VNNPs-0.

Sample	C (at%)	V (at%)	O (at%)	N (at%)
VN-0	23.65	28.18	19.69	28.49
N-CNS/VNNPs-2	27.72	26.91	16.29	29.08
N-CNS/VNNPs-1	62.62	10.25	11.22	15.91
N-CNS/VNNPs-0	83.78	1.58	5.86	8.77

**Figure 5:**
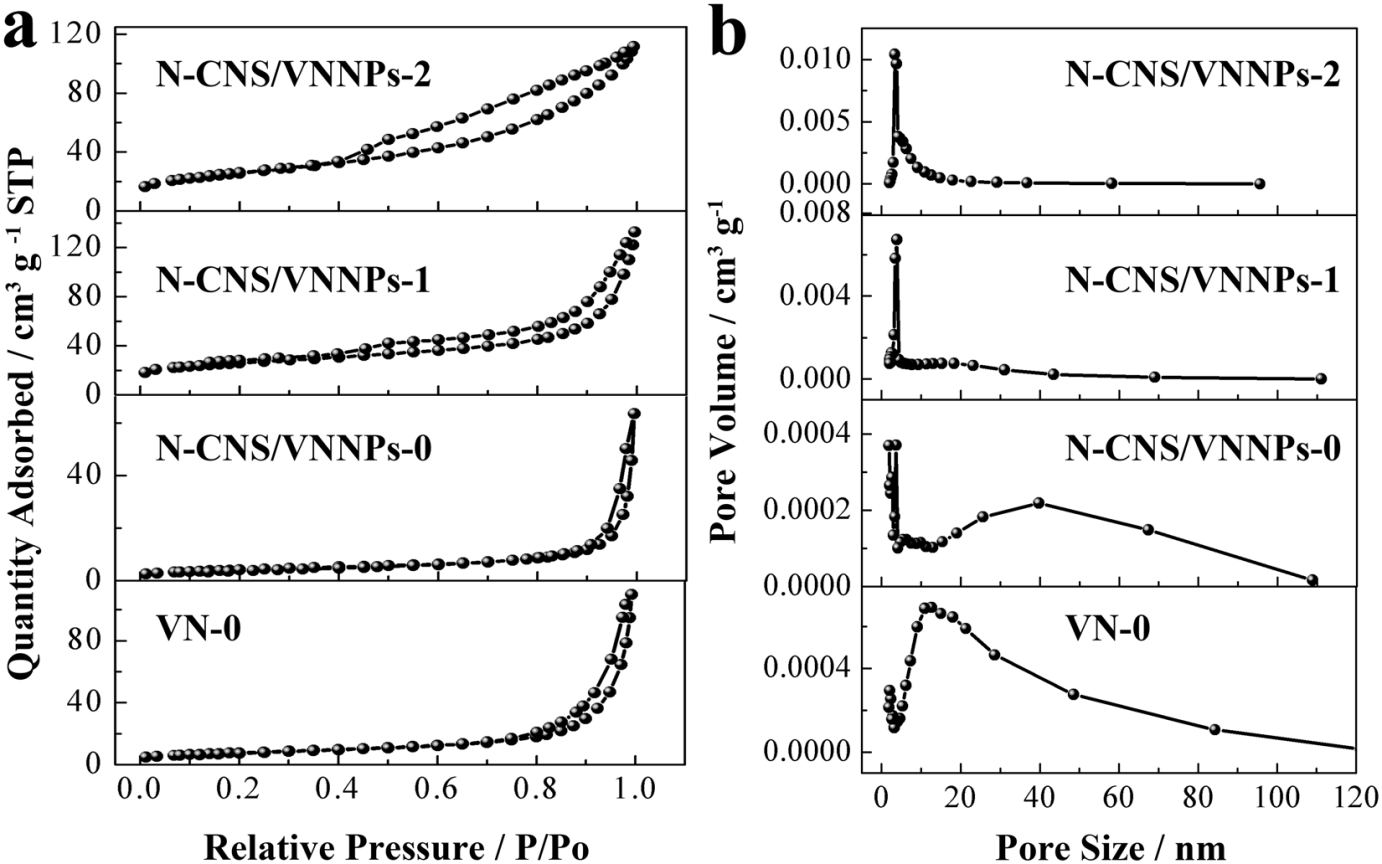
Nitrogen adsorption–desorption isotherms and pore-size distribution of VN-0, N-CNS/VNNPs-0, N-CNS/VNNPs-1, and N-CNS/VNNPs-2.

Furthermore, the surface area and pore distribution were measured using N_2_ adsorption and desorption technology, as shown in Figure 5 and Table 2. It could be found that the adsorption and desorption isotherms had a similar shape and showed not only low adsorption quantity at the relative pressure of P/P_0_ < 0.85 but also no platform at the relative pressure of P/P_0_ = 1.0. However, the hysteresis hoop indicated that there were mesopore and macropore existed in the materials (Fig. 5a). Figure 5b shows the pore size distribution of the samples. It was confirmed again that the pores included both mesopore and macropore. The detailed surface area and average pore size are shown in the Table 2. VN-0 exhibited the surface area of 27.3 m^2^ g^−1^ with the average pore size of 21.4 nm. When the pH value increased from 0 to 2 in the synthesis process, the surface area of the obtained product increased from 14.8 to 86.7 m^2^ g^−1^, and 89.5 m^2^ g^−1^ for the sample of N-CNS/VNNPs-0, N-CNS/VNNPs-1, and N-CNS/VNNPs-2, respectively. It was noted that N-CNS/VNNPs-0 showed the small sheets consisting of nanoparticles but less pores, while N-CNS/VNNPs-1 and N-CNS/VNNPs-2 showed larger sheets than that of N-CNS/VNNPs-0. However, lots of pores existed between both nanoparticles and sheet layers. In addition, it was noted that the pore size distribution of N-CNS/VNNPs-0 is wider than those of the other samples which would benefit the electrochemical performance.

**Table 2:** BET surface area and average pore size of samples

Sample	Surface area (m^2^ g^−1^)	Average pore size (nm)	Pore size distribution
VN-0	27.3	21.4	1.5~3.5, 4~100 nm
N-CNS/VNNPs-0	14.8	26.8	1.5~2.5, 2.5~10, 20~100 nm
N-CNS/VNNPs-1	86.7	10.4	1.5~20 nm
N-CNS/VNNPs-2	89.5	6.1	1.5~10 nm

**Figure 6:**
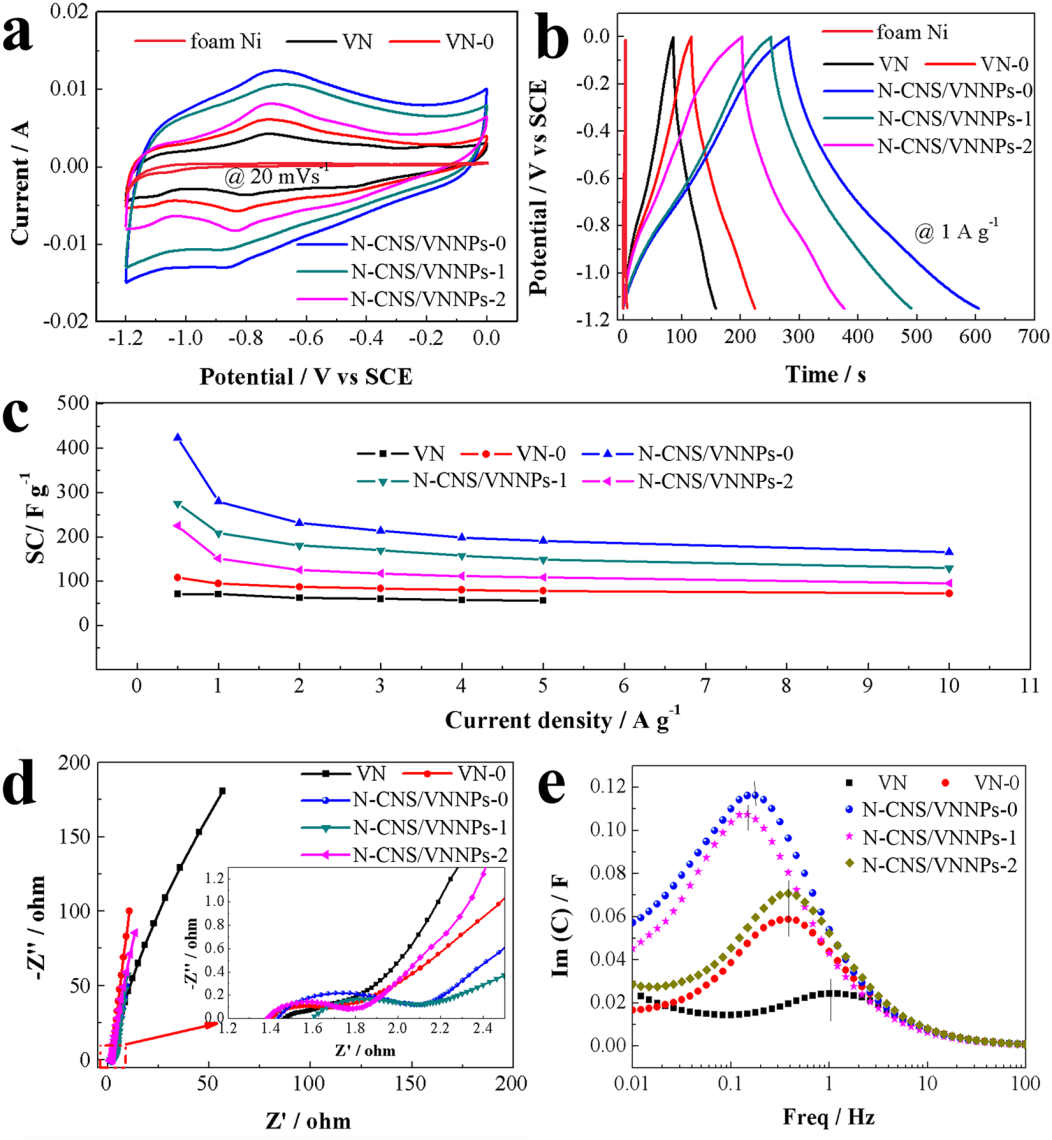
Electrochemical performance of VN, VN-0, and N-CNS/VNNPs Materials: (**a**) CV, (**b**) GCD, (**c**) SC at different current density, (**d**) Nyquist plot, and (**e**) dependence of imaginary capacitances vs. frequency.

Figure 6 shows the electrochemical performance of VN, VN-0, N-CNS/VNNPs-0, N-CNS/VNNPs-1, and N-CNS/VNNPs-2. The typical CV curves tested in the range of −1.2~0 V at scan rate of 20 mV s^−1^ exhibited a strong pair of redox peaks, indicating pseudocapacitive behavior (Fig. 6a). The specific capacitance contribution of foam Ni as the substrate was tested in the same condition. It was noted that the response current of foam Ni was very small (2.93 × 10^−4^ A) at the scan rate of 20 mV s^−1^ compared with these of the samples. Therefore, the capacitive contribution of nickel foam substrate was ignored. N-CNS/VNNPs-0 had larger CV area than these of the others’, declaring the highest specific capacitance. With the scan rate increasing, the CV shapes still remained the same without obvious change (Figures S9–13 in Supporting Information). All samples exhibited pseudocapacitive behavior and in the presence of OH^−^ ions, an equilibrium reaction occurred on the nitride or oxy-nitride surface as follows:

7 $${{\rm{VN}}}_{{\rm{x}}}{{\rm{O}}}_{{\rm{y}}}+{{\rm{OH}}}^{-}\leftrightarrow {{\rm{VN}}}_{{\rm{x}}}{{\rm{O}}}_{{\rm{y}}}{||\mathrm{OH}}^{-}+{{\rm{VN}}}_{{\rm{x}}}{{\rm{O}}}_{{\rm{y}}}\,-\,{\rm{OH}}$$

Where VN_x_O_y_||OH^−^ represented the electrical double layer formed by the hydroxyl ions adsorbed on non-specific sites, wherein a large increase in specific capacitance arose primarily due to successive oxidation by the hydroxyl species on the VN_x_O_y_ surface and electron transfer across the double layer^41^. Figure 6b shows the GCD curves ranging from −1.15 V to 0 V at current density of 1 A g^−1^, which was a reliable method of measuring the specific capacitance of the supercapacitors at constant current. The specific capacitances were calculated by using equation (1) and the specific capacitances of the as-synthesized VN, VN-0, N-CNS/VNNPs-0, N-CNS/VNNPs-1, and N-CNS/VNNPSs-2 were 70, 95, 280, 208, 151 F g^−1^, respectively. In addition, the GCD curve of foam Ni was measured. The discharging time was only 2 s and the specific capacitance was 1.6 F g^−1^. N-CNS/VNNPs-0 exhibited the highest specific capacitance of 280 F g^−1^ at 1 A g^−1^. The GCD curves of all obtained products at different current densities are also shown in Figures S9–S13 in Supporting Information, and the specific capacitance calculated based on GCD at different current densities are shown Fig. 6c. The specific capacitances of the as-synthesized VN, VN-0, N-CNS/VNNPs-0, N-CNS/VNNPs-1 and N-CNS/VNNPs-2 were 72, 108, 424, 275, 225 F g^−1^ at current density of 0.5 A g^−1^, respectively. N-CNS/VNNPs-0 showed the highest specific capacitance of 424 F g^−1^ at 0.5 A g^−1^. Even at a high current density of 10 A g^−1^, the specific capacitance of N-CNS/VNNPs-0 still remained 166 F g^−1^, which was higher than those of VN-0 (73 F g^−1^), N-CNS/VNNPs-1 (130 F g^−1^), and N-CNS/VNNPs-2 (96 F g^−1^). The results reflected that a lower pH value (high H^+^ concentration) could be beneficial for constructing the electrode material with high electrochemical performance, then the H^+^ concentration for surface-initiated *in-situ* intercalation polymerization was adjusted to 2 M. CV and GCD curves of the obtained product are shown in Figure S14 in Supporting Information. Unfortunately, the specific capacitance was calculated to be 370 F g^−1^ at 0.5 A g^−1^, which was lower than that of N-CNS/VNNPs-0. N-CNS/VNNPs-0 exhibited higher specific capacitance because of good dispersion of VN nanoparticles on the surface of N-doped carbon sheets, high N-doped level, worse crystalline structure of VN, wider pore size distribution, and such structure of 0D-2D VNNPs-N-doped carbon nanosheets. Rational amount of VN nanoparticles enhanced the capacitive contribution due to the redox reaction occurring at the surface of electrode and could shorten the pathway, easily move and touch with electroactive material surface of ions for improve specific capacitive. Meanwhile, the carbon nanosheets provided the carrier and connectivity for the VN nanoparticles due to the wide pore size distribution. Figure 6d shows the Nyquist plot and high frequency region corresponding to semicircle, reflecting the surface properties and charge transfer resistance of electrode material, and low frequency region corresponding to straight line represents ion transportation resistance^45^. The charge transfer resistance of N-CNS/VNNPs-0 (0.34 Ω) was larger than those of N-CNS/VNNPs-1 (0.24 Ω), N-CNS/VNNPs-2 (0.2 Ω), VN (0.14 Ω), and VN-0 (0.15 Ω); however, the N-CNS/VNNPs hybrids had smaller ion transportation resistance than those of VN and VN-0. In addition, such charge transfer resistance of N-CNS/VNNPs was smaller than reported in the literature^45^. Figure 6e shows the dependence of imaginary capacitances vs. frequency (Im(C) = Re(Z)/ω|Z|^2^)^55^, the relaxation time constant (τ_0_) could be calculated by the follow equation:

8 $${\tau }_{0}=\frac{1}{{{\rm{f}}}_{0}}$$

Where f_0_ is the frequency (Hz) at the position where the imaginary part of the capacitance reaches its maximum. The frequencies of VN, VN-0, N-CNS/VNNPs-0, N-CNS/VNNPs-1, N-CNS/VNNPs-2 were 1, 0.383, 0.178, 0.147 and 0.383 Hz, respectively. The relaxation time constants were 1, 2.61, 5.62, 6.80 and 2.61 s for VN, VN-0, N-CNS/VNNPs-0, N-CNS/VNNPs-1, and N-CNS/VNNPs-2, respectively.

**Figure 7:**
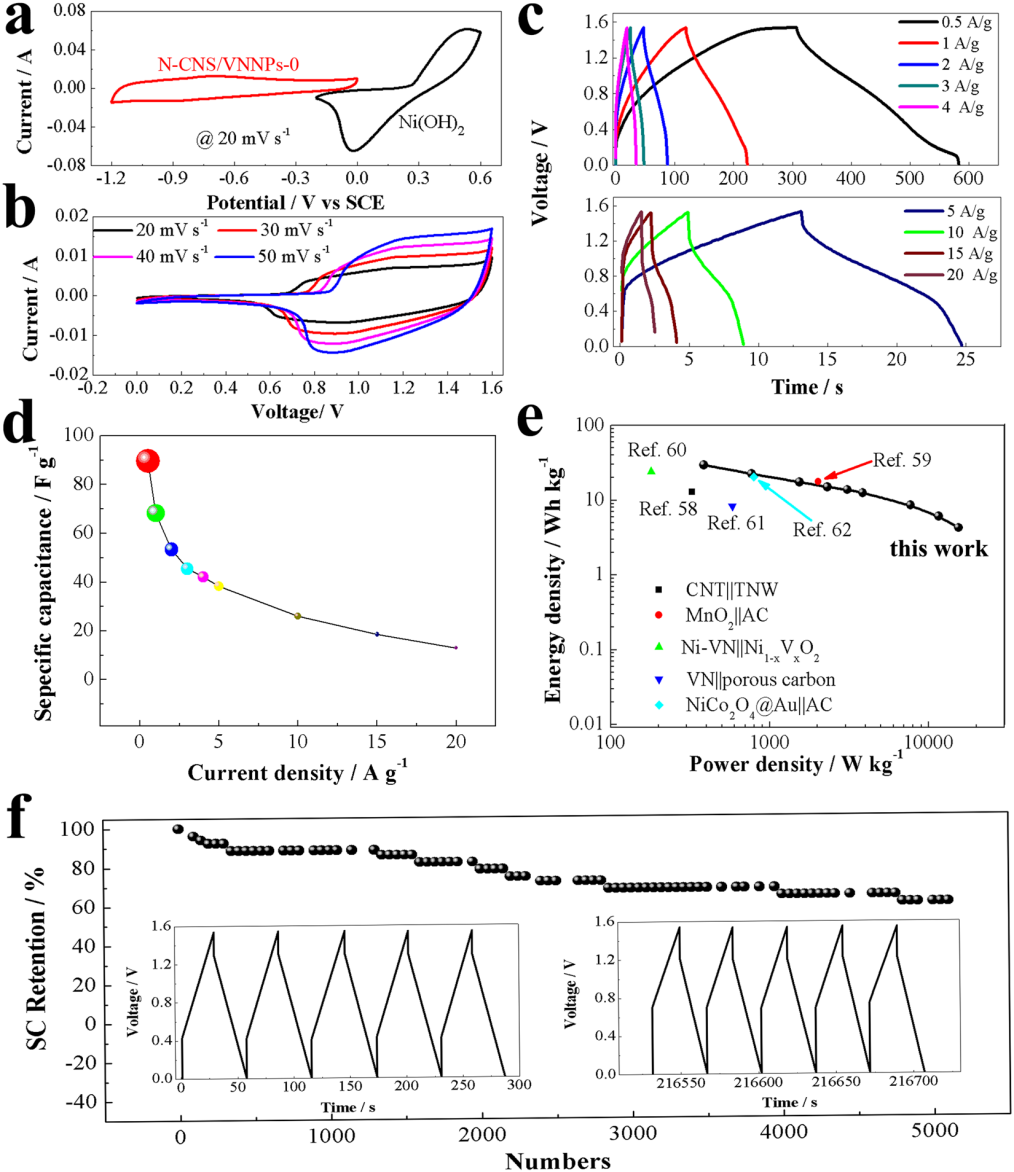
(**a**) CV curves of Ni(OH)_2_ and N-CNS/VNNPs; electrochemical performance of Ni(OH)_2_||N-CNS/VNNPs device: (**b**) CV (**c**) GCD (**d**) SC at different current density from 0.5 to 20 A g^−1^, (**e**) Ragone plot, and (**f**) cycle life at current density of 2.7 A g^−1^ (inset: GCD curves for the first five cycle and last five cycle).

The CV curves of Ni(OH)_2_ and N-CNS/VNNPs were shown in Fig. 7a. Based on the CV curves, all of Ni(OH)_2_ and N-CNS/VNNPs electrodes exhibited pesudocapacitive behavior. The asymmetric supercapacitor device of Ni(OH)_2_||N-CNS/VNNPs was assembled by using N-CNS/VNNPs as the negative electrode and Ni(OH)_2_ as the positive electrode. The electrode performance of the device was tested in 2 M KOH and the results are shown in Fig. 7b–f. Both CV curves at scan rates from 20 to 50 mV s^−1^ and GCD curves at current densities from 0.5 to 20 A g^−1^ exhibited pseudocapacitive behavior (Fig. 7b and c). The highest specific capacitance was delivered to be 89.6 F g^−1^ at current density of 0.5 A g^−1^, and the specific capacitance still maintained 13 F g^−1^ even when the current density increased to 20 A g^−1^ (40 times of initial current density) **(**Fig. 7d**)**. Figure S15 in Supporting Information shows the dependence of imaginary capacitances vs. frequency for the Ni(OH)_2_||N-CNS/VNNPs device, and the relaxation time constant was calculated to be τ_0_ = 10 s by using equation 7. This is similar to that of carbon||carbon supercapacitors^56^, indicating pretty good current response of the prepared Ni(OH)_2_||N-CNS/VNNPs asymmetric supercapacitor device. As Ragone plots shown in Fig. 7e, the energy density and power density were calculated using galvanostatic charging-discharging data obtained at different current densities. The device delivered a maximum energy density of 29.5 Wh kg^−1^ with the power density of 385 W kg^−1^, and a maximum power density of 15.4 kW kg^−1^ with the energy density of 4.28 Wh kg^−1^. Compared with the other reported supercapacitor devices, such as carbon||carbon device (4.4~12.6 Wh kg^−1^)^57^, carbon nanotube||TiO_2_ nanowire (12.5 Wh kg^−1^)^58^, MnO_2_||AC (17 Wh kg^−1^)^59^, Ni-VN||Ni_1−x_V_x_O_2_ (23.3 Wh kg^−1^)^60^, VN||porous carbon (8 Wh kg^−1^)^61^ and NiCo_2_O_4_||AC (19.56 Wh kg^−1^)^62^, the P_m_ − E_m_ curve of our as-prepared asymmetric supercapacitor was entirely in the top right corner of the Ragone plot, confirming the superior energy storage ability of our asymmetric supercapacitor sample. In addition, the cycle stability was also measured for practical application. As shown in Fig. 7f, the cycle life was tested at the current density of 2.7 A g^−1^, and after 5000 cycles, the specific supercapacitor retention was 60%, suggesting good cycle stability. In addition, cycle stability was also measured at higher density of 6 A g^−1^, as shown in Figure S16 in the Supporting Information. After 11, 000 cycle numbers, the specific capacitance remained 95.6% of the initial specific capacitance and then going on circulation at the current density of 3 A g^−1^, specific capacitance retention was keeping 86.5%. The higher decrement at lower current density of 3 A g^−1^, that was because the depth of redox reaction was deeper than that of higher current density of 6 A g^−1^. In general, the superior performance of the Ni(OH)_2_||N-CNS/VNNPs asymmetry supercapacitor was mainly due to the advanced structure of the electroactive material of N-CNS/VNNPs: i) the unique structure architecture consisted of 2D N-doped carbon nanosheets and 0D VN nanoparticles, which reduced the diffusion length of the ions and electrons within the electrode phase; ii) 2D N-doped carbon nanosheets provided electrochemical active site and protect 0D VN nanoparticles during charging-discharging process; and iii) the synergistic effect between the two components of the composite.

## Conclusions

N-doped carbon nanosheets/vanadium nitride nanoparticles (N-CNS/VNNPs) hybrids were synthesized via a novel method of combining surface-initiated *in-situ* intercalative polymerization and thermal-treatment, which made N-CNS/VNNPs include 2D carbon nanosheets and 0D VN nanoparticles. N-CNS/VNNPs-0 showed higher specific capacitance of 424 F g^−1^ than these of N-CNS/VNNPs-1, N-CNS/VNNPs-2, and pristine VN at current density of 0.5 A g^−1^. The asymmetric energy device of Ni(OH)_2_||N-CNS/VNNPs delivered a maximum energy density of 29.5 Wh kg^−1^ with the power density of 385 W kg^−1^, and maximum power density of 15.4 kW kg^−1^ with the energy density of 4.28 Wh kg^−1^ and retained 60% specific capacitance at 2.7 A g^−1^ after 5000 cycles.

## Electronic supplementary material

Supporting information

## References

[1] Wang Y, Song Y, Xia Y (2016). Electrochemical capacitors: mechanism, materials, systems, characterization and applications. Chemical Society Reviews.

[2] Simon P, Gogotsi Y (2008). materials for electrochemical capacitor. Nature Materials.

[3] Miller JR, Burke AF (2008). Electrochemical Capacitors Challenges and Opportunities for Real-World Applications. The Electrochemical Society Interface.

[4] Burke A (2000). Ultracapacitors: why, how, and where is the technology. Journal of Power Sources.

[5] Yan J, Wang Q, Wei T, Fan Z (2014). Recent Advances in Design and Fabrication of Electrochemical Supercapacitors with High Energy Densities. Advanced Energy Materials.

[6] Kimizuka O (2008). Electrochemical doping of pure single-walled carbon nanotubes used as supercapacitor electrodes. Carbon.

[7] Shi Q (2015). Nitrogen-doped ordered mesoporous carbons based on cyanamide as the dopant for supercapacitor. Carbon.

[8] Xia K, Gao Q, Jiang J, Hu J (2008). Hierarchical porous carbons with controlled micropores and mesopores for supercapacitor electrode materials. Carbon.

[9] Wei T, Wei X, Gao Y, Li H (2015). Large scale production of biomass-derived nitrogen-doped porous carbon materials for supercapacitors. Electrochimica Acta.

[10] Kang D (2015). “Egg-Box”-Assisted Fabrication of Porous Carbon with Small Mesopores for High-Rate Electric Double Layer Capacitors. ACS Nano.

[11] Biswal M, Banerjee A, Deo M, Ogale S (2013). From dead leaves to high energy density supercapacitors. Energy & Environmental Science.

[12] Zhao L (2010). Nitrogen-Containing Hydrothermal Carbons with Superior Performance in Supercapacitors. Advanced Materials.

[13] Hulicova-Jurcakova D (2009). Nitrogen-Enriched Nonporous Carbon Electrodes with Extraordinary Supercapacitance. Advanced Functional Materials.

[14] Zhang LL, Zhou R, Zhao XS (2010). Graphene-based materials as supercapacitor electrodes. Journal of Materials Chemistry.

[15] Kang XJ, Zhang JM, Sun XW, Zhang FR, Zhang YX (2016). One-pot synthesis of vanadium dioxide nanoflowers on graphene oxide. Ceramics International.

[16] Kudo T (2002). Amorphous V_2_O_5_/carbon composites as electrochemical supercapacitor electrodes. Solid State Ionics.

[17] Wang JG, Kang F, Wei B (2015). Engineering of MnO_2_-based nanocomposites for high-performance supercapacitors. Progress in Materials Science.

[18] Huang M, Li F, Dong F, Zhang YX, Zhang LL (2015). MnO_2_-based nanostructures for high-performance supercapacitors. Journal of Materials Chemistry. A.

[19] Huang M (2014). Layered manganese oxides-decorated and nickel foam-supported carbon nanotubes as advanced binder-free supercapacitor electrodes. Journal of Power Sources.

[20] Kong LB, Lang JW, Liu M, Luo YC, Kang L (2009). Facile approach to prepare loose-packed cobalt hydroxide nano-flakes materials for electrochemical capacitors. Journal of Power Sources.

[21] Yuan C (2012). Growth of ultrathin mesoporous Co_3_O_4_*nanosheet arrays on Ni foam for h*igh-performance electrochemical capacitors. Energy & Environmental Science.

[22] Tan YT, Liu Y, Kong LB, Kang L, Ran F (2017). Supercapacitor electrode of nano-Co_3_O_4_ decorated with gold nanoparticles via *in-situ* reduction method. Journal of Power Sources.

[23] Xiang C, Li M, Zhi M, Manivannan A, Wu N (2013). A reduced graphene oxide/Co_3_O_4_ composite for supercapacitor electrode. Journal of Power Sources.

[24] Sk MM, Yue CY, Ghosh K, Jena RK (2016). Review on advances in porous nanostructured nickel oxides and their composite electrodes for high-performance supercapacitors. Journal of Power Sources.

[25] Zhang X (2010). Synthesis of porous NiO nanocrystals with controllable surface area and their application as supercapacitor electrodes. Nano Research.

[26] Li B, Zheng M, Xue H, Pang H (2016). High performance electrochemical capacitor materials focusing on nickel based materials. Inorganic Chemistry Frontiers.

[27] Lang, J. W., Kong, L. B., Wu, W. J., Luo, Y. C. & Kang, L. Facile approach to prepare loose-packed NiO nano-flakes materials for supercapacitors. *Chemical Communications*, 4213–4215 (2008). 10.1039/b800264a 18802533

[28] Lang JW (2008). A facile approach to the preparation of loose-packed Ni(OH)_2_ nanoflake materials for electrochemical capacitors. Journal of Solid State Electrochemistry.

[29] Lang JW, Kong LB, Liu M, Luo YC, Kang L (2009). Asymmetric supercapacitors based on stabilized α-Ni(OH)2 and activated carbon. Journal of Solid State Electrochemistry.

[30] Lang JW, Kong LB, Wu WJ, Luo YC, Kang L (2009). Synthesis, characterization, and electrochemical properties of Ni(OH)_2_/ultra-stable Y zeolite composite. Journal of Materials Science.

[31] Zhang Y, Wu J, Zheng TX, Zhang YX, Liu H (2016). Binder-free supercapacitive of ultrathin Co(OH)_2_ nanosheets-decorated nitrogen-doped carbon nanotubes core-shell nanostructures. Materials Technology.

[32] Kong LB (2010). Co(OH)_2_/SBA-15 molecular sieves nanocomposite materials for electrochemical capacitors. Materials Chemistry and Physics.

[33] Wang YG, Li HQ, Xia YY (2006). Ordered Whiskerlike Polyaniline Grown on the Surface of Mesoporous Carbon and Its Electrochemical Capacitance Performance. Advanced Materials.

[34] Fan LZ (2007). High Electroactivity of Polyaniline in Supercapacitors by Using a Hierarchically Porous Carbon Monolith as a Support. Advanced Functional Materials.

[35] Zhang J, Kong LB, Wang B, Luo YC, Kang L (2009). *In-situ* electrochemical polymerization of multi-walled carbon nanotube/polyaniline composite films for electrochemical supercapacitors. Synthetic Metals.

[36] Tan YT, Ran F, Kong LB, Liu J, Kang L (2012). Polyaniline nanoparticles grown on the surface of carbon microspheres aggregations for electrochemical supercapacitors. Synthetic Metals.

[37] Zhang J, Kong LB, Li H, Luo YC, Kang L (2010). Synthesis of polypyrrole film by pulse galvanostatic method and its application as supercapacitor electrode materials. Journal of Materials Science.

[38] Zhong Y (2016). Transition Metal Carbides and Nitrides in Energy Storage and Conversion. Advanced Science.

[39] Balogun MS (2015). Recent advances in metal nitrides as high-performance electrode materials for energy storage devices. Journal of Materials Chemistry A.

[40] Bouhtiyya, S. *et al*. Transition Metal Nitrides Thin Films for Supercapacitor Applications. *Meeting Abstracts* **MA2012-02**, 494 (2012).

[41] Choi D, Blomgren GE, Kumta PN (2006). Fast and Reversible Surface Redox Reaction in Nanocrystalline Vanadium Nitride Supercapacitors. Advanced Materials.

[42] Zhou X, Chen H, Shu D, He C, Nan J (2009). Study on the electrochemical behavior of vanadium nitride as a promising supercapacitor material. Journal of Physics and Chemistry of Solids.

[43] Glushenkov AM, Hulicova-Jurcakova D, Llewellyn D, Lu GQ, Chen Y (2010). Structure and Capacitive Properties of Porous Nanocrystalline VN Prepared by Temperature-Programmed Ammonia Reduction of V_2_O_5_. Chemistry of Materials.

[44] Lu X (2013). High Energy Density Asymmetric Quasi-Solid-State Supercapacitor Based on Porous Vanadium Nitride Nanowire Anode. Nano Letters.

[45] Xu Y, Wang J, Shen L, Dou H, Zhang X (2015). One-Dimensional Vanadium Nitride Nanofibers Fabricated by Electrospinning for Supercapacitors. Electrochimica Acta.

[46] Wu Y, Ran F (2017). Vanadium nitride quantum dot/nitrogen-doped microporous carbon nanofibers electrode for high-performance supercapacitors. Journal of Power Sources.

[47] Balamurugan J, Karthikeyan G, Tran Duy T, Kim NH, Lee JH (2016). Facile synthesis of vanadium nitride/nitrogen-doped graphene composite as stable high performance anode materials for supercapacitors. Journal of Power Sources.

[48] Wang L, Sun J, Song R, Yang S, Song H (2016). Hybrid 2D-0D Graphene-VN Quantum Dots for Superior Lithium and Sodium Storage. Advanced Energy Materials.

[49] Park N (2002). Synthesis and electrochemical properties of V_2_O_5_ intercalated with binary polymers. Journal of Power Sources.

[50] Liracantu M, Gomezromero P (1999). Synthesis and Characterization of Intercalate Phases in the Organic–Inorganic Polyaniline/V_2_O_5_ System. Journal of Solid State Chemistry.

[51] Tran HD (2011). The oxidation of aniline to produce “polyaniline”: a process yielding many different nanoscale structures. Journal of Materials Chemistry.

[52] Li G, Jiang L, Peng H (2007). One-Dimensional Polyaniline Nanostructures with Controllable Surfaces and Diameters Using Vanadic Acid as the Oxidant. Macromolecules.

[53] Li G, Zhang C, Peng H, Chen K (2009). One-Dimensional V_2_O_5_@Polyaniline Core/Shell Nanobelts Synthesized by an *In situ* Polymerization Method. Macromolecular Rapid Communications.

[54] Lin T (2015). Nitrogen-doped mesoporous carbon of extraordinary capacitance for electrochemical energy storage. Science.

[55] Zhang SL, Pan N (2015). Supercapacitors Performance Evaluation. Advanced Energy Materials.

[56] Taberna PL, Simon P, Fauvarque JF (2003). Electrochemical Characteristics and Impedance Spectroscopy Studies of Carbon-Carbon Supercapacitors. Journal of The Electrochemical Society.

[57] Bichat MP, Raymundo-Pinero E, Beguin F (2010). High voltage supercapacitor built with seaweed carbons in neutral aqueous electrolyte. Carbon.

[58] Wang Q, Wen ZH, Li JH (2006). A Hybrid Supercapacitor Fabricated with a Carbon Nanotube Cathode and a TiO_2_–B Nanowire Anode. Advanced Functional Materials.

[59] Qu Q (2009). Electrochemical Performance of MnO_2_ Nanorods in Neutral Aqueous Electrolytes as a Cathode for Asymmetric Supercapacitors. The Journal of Physical Chemistry C.

[60] Ji C, Bi J, Wang S, Zhang X, Yang S (2016). Ni nanoparticle doped porous VN nanoflakes assembled into hierarchical hollow microspheres with a structural inheritance from the Ni_1−x_V_x_O_2_ cathode material for high performance asymmetric supercapacitors. Journal of Materials Chemistry A.

[61] Yang Y (2017). Novel Hybrid Nanoparticles of Vanadium Nitride/Porous Carbon as an Anode Material for Symmetrical Supercapacitor. Nano-Micro Letters.

[62] Zhu J, Xu Z, Lu B (2014). Ultrafine Au nanoparticles decorated NiCo_2_O_4_ nanotubes as anode material for high-performance supercapacitor and lithium-ion battery applications. Nano Energy.

