# Author Correction: Concise N-doped Carbon Nanosheets/Vanadium Nitride Nanoparticles Materials via Intercalative Polymerization for Supercapacitors

**DOI:** 10.1038/s41598-018-24109-4

**Published:** 2018-04-18

**Authors:** Yongtao Tan, Ying Liu, Zhenghua Tang, Zhe Wang, Lingbin Kong, Long Kang, Zhen Liu, Fen Ran

**Affiliations:** 1 State Key Laboratory of Advanced Processing and Recycling of Non-ferrous Metals, Lanzhou University of Technology, Lanzhou, 730050 P. R. China; 2 School of Material Science and Engineering, Lanzhou University of Technology, Lanzhou, 730050 Gansu P. R. China; 3 Department of Physics & Engineering, Frostburg State University, Frostburg, MD 21532-2303 USA; 4 Guangzhou Key Laboratory for Surface Chemistry of Energy Materials, New Energy Research Institute, School of Environment and Energy, South China University of Technology, Guangzhou Higher Education Mega Centre, Guangzhou, 510006 China; 5 Guangdong Provincial Key Laboratory of Atmospheric Environment and Pollution Control, Guangdong Provincial Engineering and Technology Research Center for Environmental Risk Prevention and Emergency Disposal, South China University of Technology, Guangzhou Higher Education Mega Centre, Guangzhou, 510006 China; 6 Department of Chemistry, Xavier University of Louisiana, New Orleans, LA 70125 USA

Correction to: *Scientific Reports* 10.1038/s41598-018-21082-w, published online 13 February 2018

The Acknowledgements section in this Article is incomplete:

“This work was partly supported by the National Natural Science Foundation of China (51203071, 51363014, 51463012, and 51763014), China Postdoctoral Science Foundation (2014M552509, and 2015T81064), Natural Science Funds of the Gansu Province (1506RJZA098), and the Program for Hongliu Distinguished Young Scholars in Lanzhou University of Technology (J201402).”

should read:

“This work was partly supported by the National Natural Science Foundation of China (51203071, 51363014, 51463012, and 51763014), China Postdoctoral Science Foundation (2014M552509, and 2015T81064), Natural Science Funds of the Gansu Province (1506RJZA098), and the Program for Hongliu Distinguished Young Scholars in Lanzhou University of Technology (J201402). Dr. Zhe Wang would like to acknowledge the support from the National Science Foundation (Grant 1700429) and NIMHD-RCMI (5G12MD007595) and the NIGMS-BUILD (8UL1GM118967) and this publication was also made possible by the Louisiana Cancer Research Consortium.”

