# Supplementary material for: Concise N-doped Carbon Nanosheets/Vanadium Nitride Nanoparticles Materials via Intercalative Polymerization for Supercapacitors
Source: Sci Rep. 2018 Feb 13;8:2915. doi: 10.1038/s41598-018-21082-w (PMC5811484; doi:10.1038/s41598-018-21082-w)
Supplement: Supplementary file 1 — Supporting information [file 41598_2018_21082_MOESM1_ESM.pdf]

Supporting Information

Concise N-doped Carbon Nanosheets/Vanadium Nitride Nanoparticles Materials via Intercalative Polymerization for Supercapacitors

Yongtao Tan [a,b], Ying Liu [a,b], Zhenghua Tang[d,e], Zhe Wang[f], Lingbin Kong [a,b], Long Kang [a,b], Zhen Liu[c*], Fen Ran [a,b,*]

[a] *State Key Laboratory of Advanced Processing and Recycling of Non-ferrous Metals, Lanzhou University of Technology, Lanzhou 730050, P. R. China*

[b] *School of Material Science and Engineering, Lanzhou University of Technology, Lanzhou 730050, Gansu, P. R. China*

[c] *Department of Physics & Engineering, Frostburg State University, Frostburg, MD 21532-2303, USA*

[d] *Guangzhou Key Laboratory of Energy Materials Surface Chemistry, New Energy Research Institute, School of Environment and Energy, South China University of Technology, Guangzhou Higher Education Mega Centre, Guangzhou, 510006, China.*

[e] *Guangdong Provincial Key Laboratory of Atmospheric Environment and Pollution Control, Guangdong Provincial Engineering and Technology Research Center for Environmental Risk Prevention and Emergency Disposal, South China University of Technology, Guangzhou Higher Education Mega Centre, Guangzhou, 510006, China*

[f] *Department of Chemistry, Xavier University of Louisiana, New Orleans, LA 70125, USA*

*Corresponding author: Fen Ran (ranfen@163.com) or Zhen Liu (zliu@frostburg.edu)

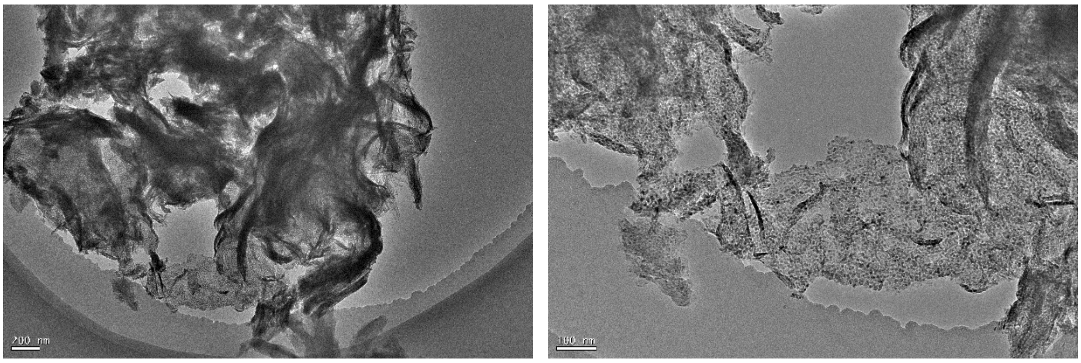

Figure S1 TEM of N-CNS/VNNPs-0

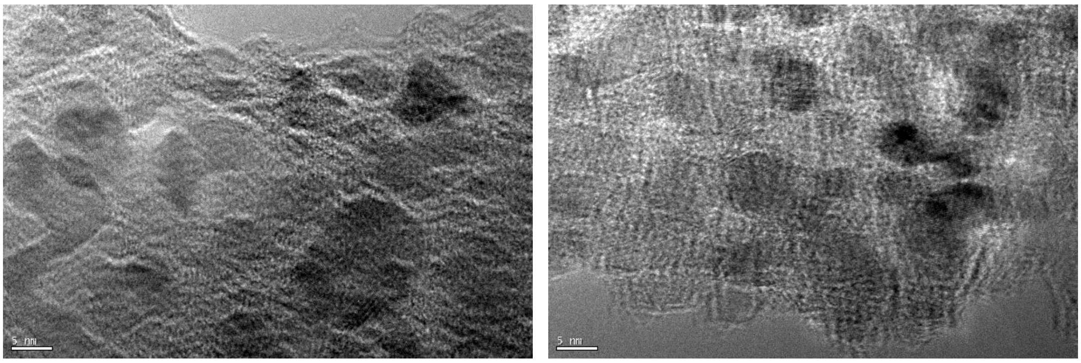

Figure S2 HRTEM of N-CNS/VNNPs-0

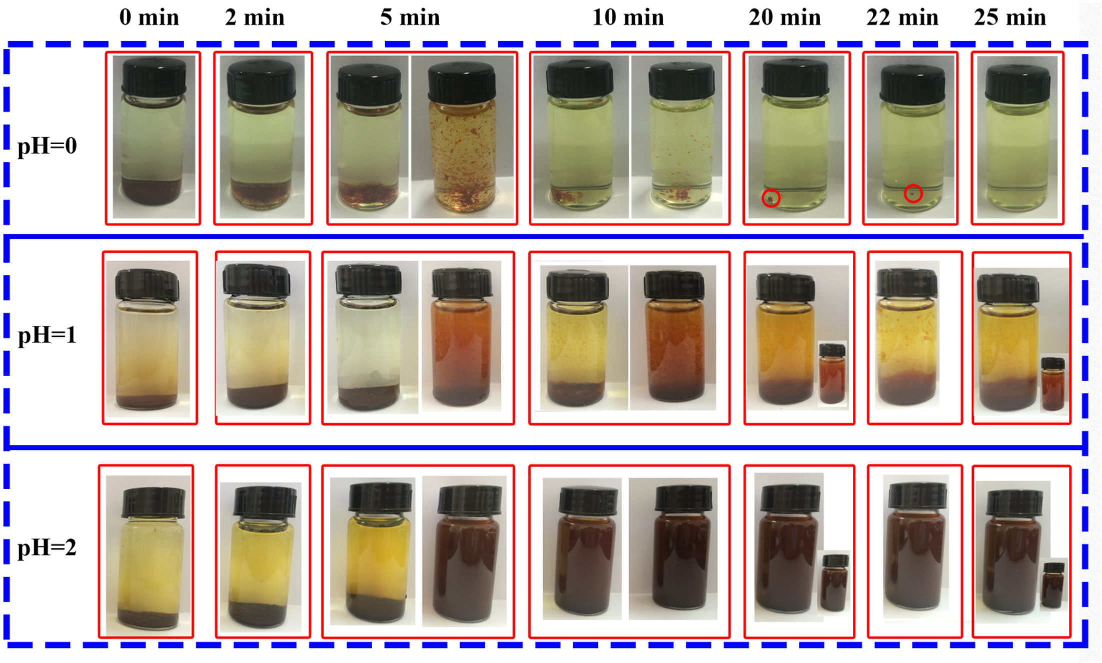

Figure S3 *the photos of samples in different ultrasonic time*

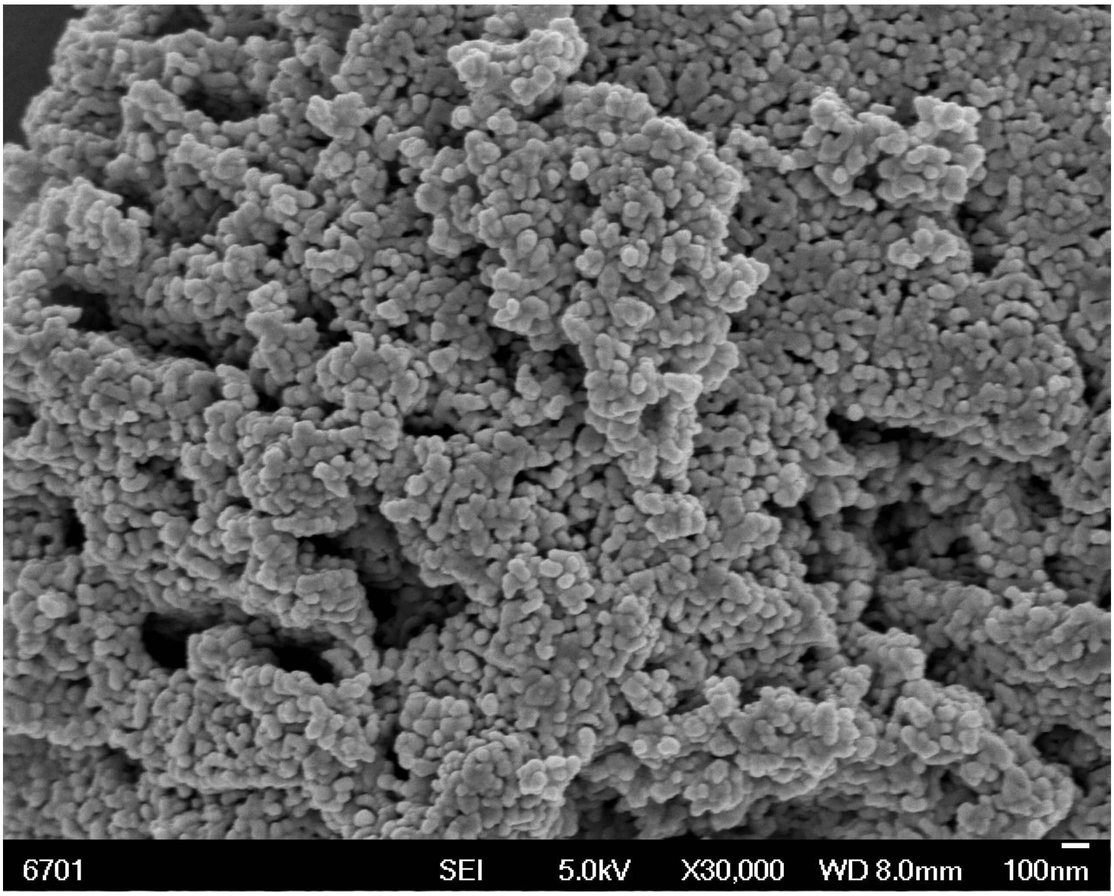

Figure S4 SEM images of VN based on NH_4VO_3

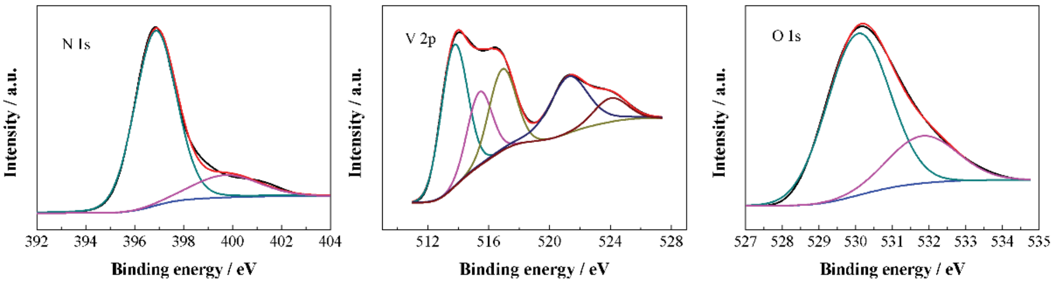

Figure S5 High resolution XPS spectra of VN-0: N 1s, V 2p and O 1s

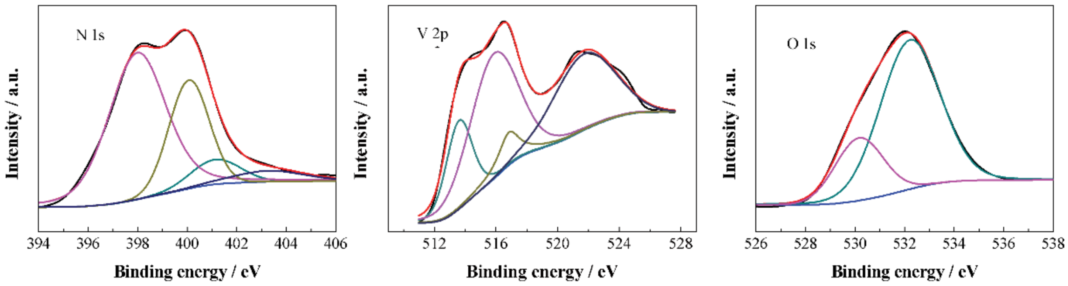

Figure S6 High resolution XPS spectra of N-CNS/VNNPs-0: N 1s, V 2p and O 1s

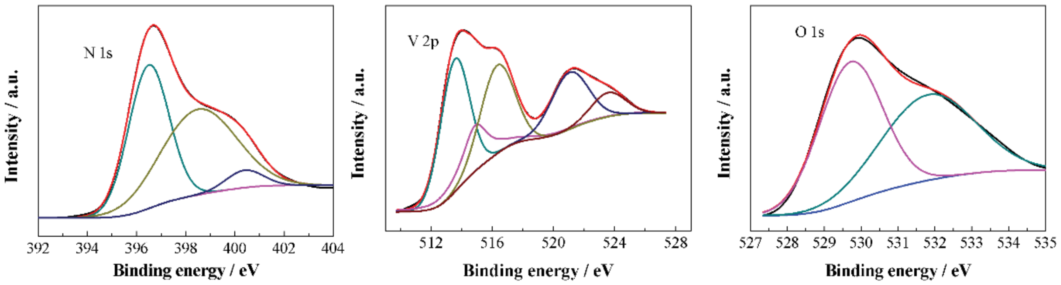

Figure S7 High resolution XPS spectra of N-CNS/VNNPs-1: N 1s, V 2p and O 1s

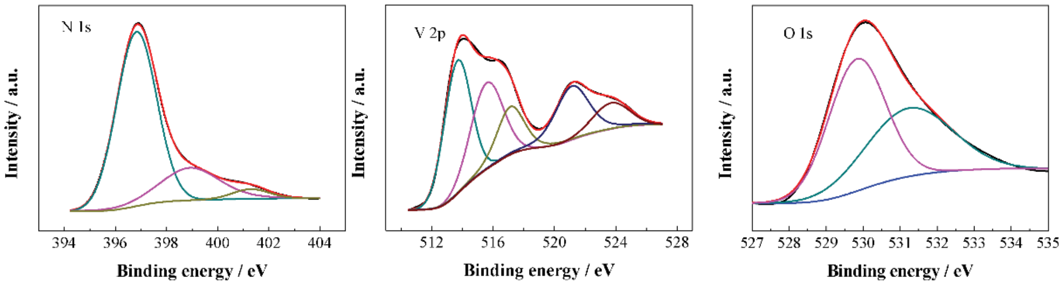

Figure S8 High resolution XPS spectra of N-CNS/VNNPs-2: N 1s, V 2p and O 1s

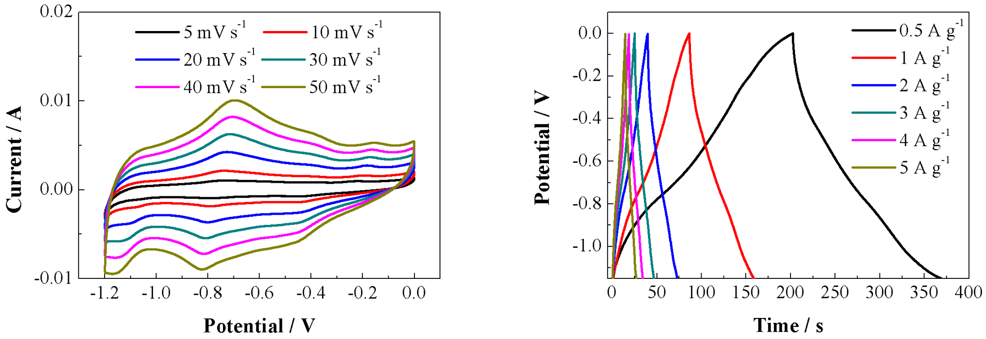

Figure S9 CV and GCD of VN

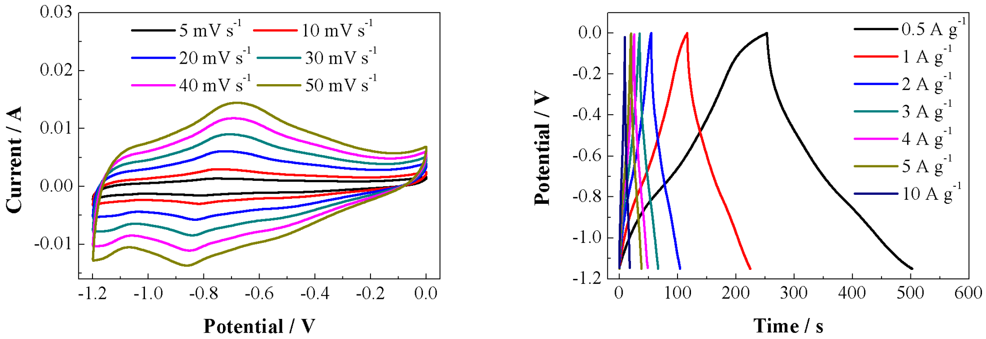

Figure S10 CV and GCD of VN-0

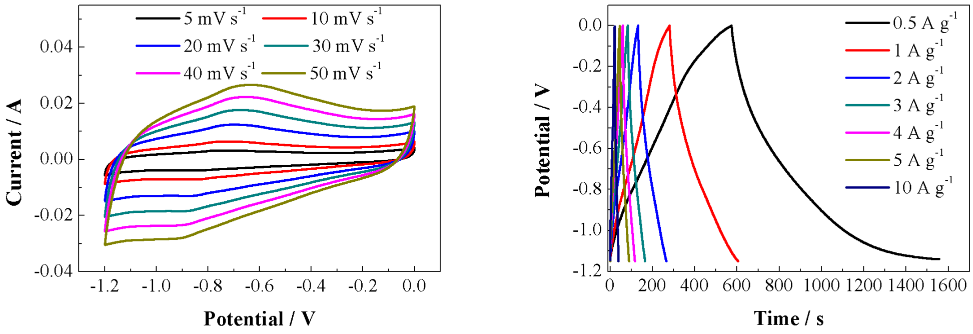

Figure S11 CV and GCD of N-CNS/VNNPs-0

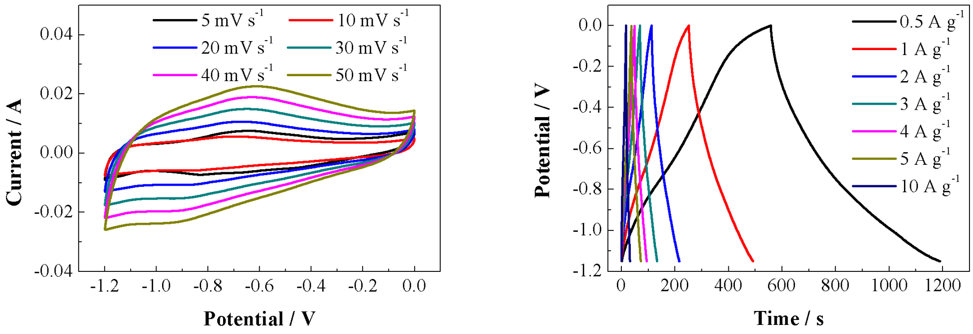

Figure S12 CV and GCD of N-CNS/VNNPs-1

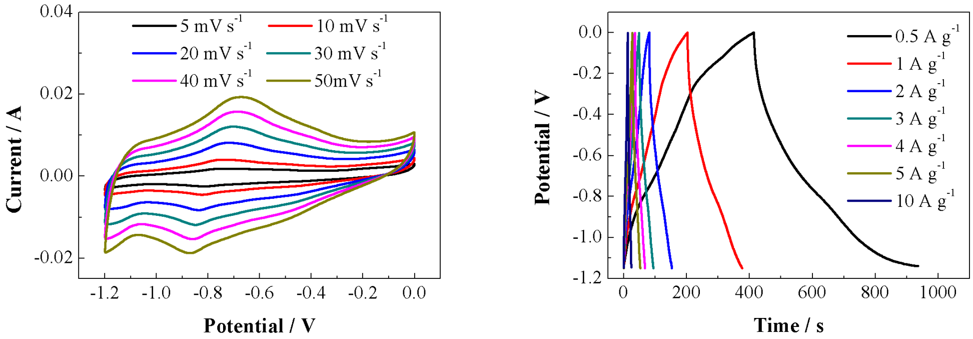

Figure S13 CV and GCD of N-CNS/VNNPs-2

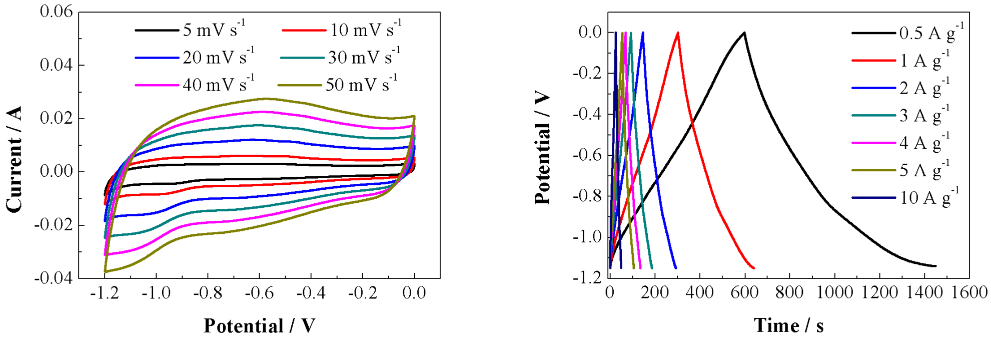

Figure S14 CV and GCD of N-CNS/VNNPs composite in 2 M HCl

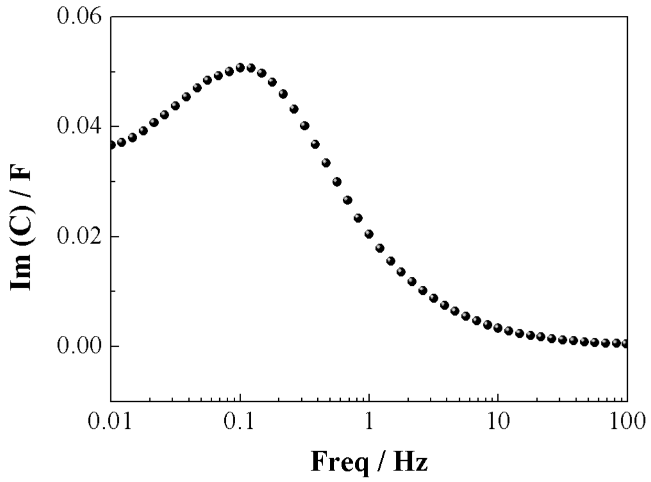

Figure S15 Dependence of imaginary capacitances vs. frequency for $Ni(OH)_2$//N-CNS/VNNPs

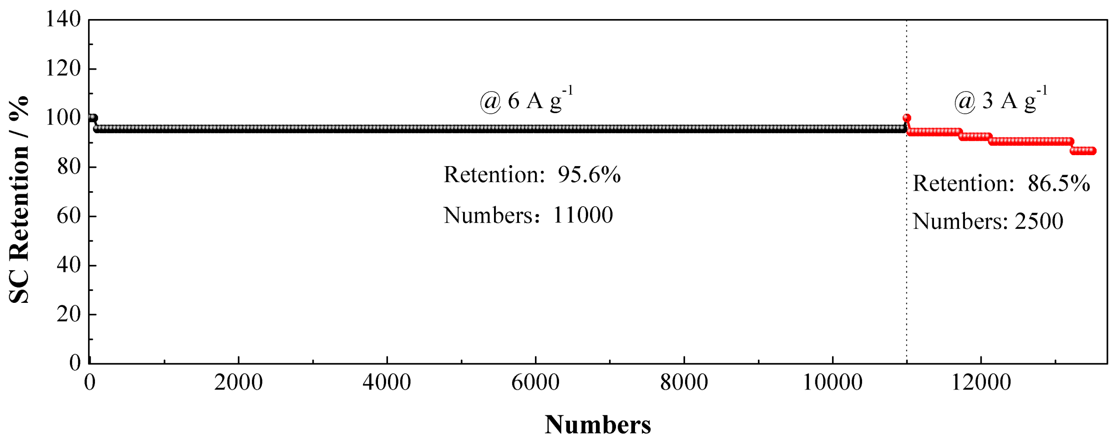

Figure S16 *Cycle life of $Ni(OH)_2$||N-CNS/VNNPs at current density of 6 A g^{-1} and that at 3 A g^{-1}*
